# *Staphylococcus aureus* Extracellular Vesicles Enhance PslE‐Mediated Pathogenesis in *Pseudomonas aeruginosa*

**DOI:** 10.1002/mbo3.70114

**Published:** 2026-01-19

**Authors:** Phawinee Subsomwong, Rojana Sukchawalit, Naoko Watabe, Akio Nakane, Krisana Asano

**Affiliations:** ^1^ Department of Microbiology and Immunology Hirosaki University Graduate School of Medicine Hirosaki Aomori Japan; ^2^ Laboratory of Biotechnology, Chulabhorn Research Institute Bangkok Thailand; ^3^ Department of Biopolymer and Health Science Hirosaki University Graduate School of Medicine Hirosaki Japan

**Keywords:** biofilm formation, extracellular vesicles, *P. aeruginosa* uptake, Psl exopolysaccharide polymerization, *Pseudomonas aeruginosa*, *Staphylococcus aureus*

## Abstract

Coinfection of *Pseudomonas aeruginosa* (*P. aeruginosa*) and *Staphylococcus aureus* (*S. aureus*) is frequently observed. Our previous study demonstrated that *S. aureus*‐derived extracellular vesicles (SaEVs) promote *P. aeruginosa* pathogenicity by increasing lipopolysaccharide (LPS) production, promoting biofilm formation and decreasing the uptake of *P. aeruginosa* by macrophages. Proteomic analysis revealed that SaEVs enhance the production of PslE, an exopolysaccharide biosynthetic protein in *P. aeruginosa*, but the role of Psl exopolysaccharide polymerization on SaEV‐mediated *P. aeruginosa* pathogenicity is unclear. In this study, a *pslE*‐deletion mutant of *P. aeruginosa* (PaΔ*pslE*) was constructed, and the effect of SaEVs on the pathogenicity of this mutant was evaluated. Our results showed that SaEVs significantly increased the expression of *pslA*, *E*, *J*, *K*, and *L* genes in the *psl* cluster of *P. aeruginosa* wildtype (PaWT), and this effect was abolished in PaΔ*pslE*. In addition, LPS production and biofilm formation were reduced in PaΔ*pslE* compared to PaWT. SaEVs significantly enhanced LPS production and biofilm formation in PaWT. On the other hand, the effects of SaEVs on the production of lipid A and LPS core and biofilm formation in PaΔ*pslE* were abolished. Invasion of PaWT and PaΔ*pslE* into HaCaT human epithelial cells was not significantly different and the effect of SaEVs on these bacterial cell invasions was not found. However, the uptake of SaEV‐treated PaWT by macrophages significantly reduced compared to nontreated PaWT, whereas SaEVs did not alter the uptake of PaΔ*pslE*. These results suggest that PslE is required for SaEV‐mediated *P. aeruginosa* pathogenicity. SaEVs upregulate *pslE* gene as well as other exopolysaccharide polymerization‐related genes, increase LPS production and biofilm formation, and affect the uptake of *P. aeruginosa* by macrophages.

## Graphical abstract

PslE is essential for the ability of *S. aureus*‐derived extracellular vesicles to enhance *P. aeruginosa* pathogenicity.

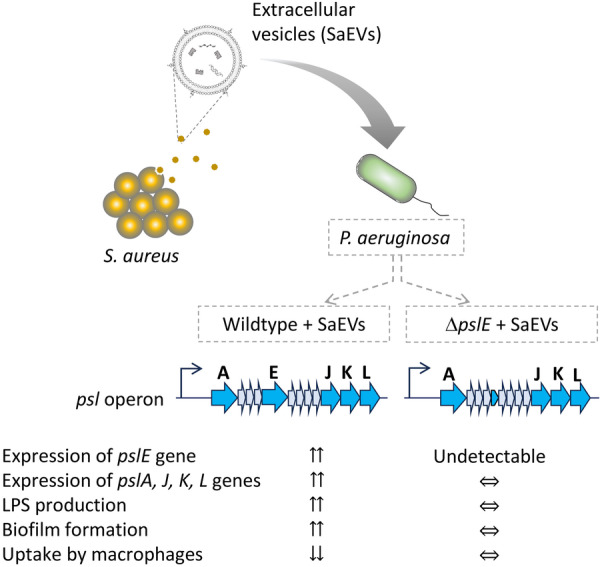

**Abbreviations**
CF: cystic fibrosis
DMEM: Dulbecco's Eagle's minimum essential medium
FBS: fetal bovine serum
Gm: gentamicin
LB: Luria–Bertani
LPS: lipopolysaccharide
OD: optical density
PaWT: *P. aeruginosa* wildtype
PaΔ*pslE*
: *pslE* gene‐deletion mutant of *P. aeruginosa*
PBS: phosphate‐buffered saline
Pel: pellicle
Psl: polysaccharide synthesis locus
SaEVs: *S. aureus* extracellular vesicles
TSA: Tryptic soy agar
TSB: Tryptic soy broth

## 1 Introduction

*Pseudomonas aeruginosa* (*P*. *aeruginosa*) and *Staphylococcus aureus* (*S. aureus*) are opportunistic pathogens that often coexist in various conditions, including in chronic wounds or cystic fibrosis (CF) lungs (Bernardy et al. 2022; DeLeon et al. 2014; Fugère et al. 2014). Longitudinal culture positivity for *S. aureus* and *P. aeruginosa* in children and adults with CF revealed that coinfection with these pathogens is common and increased from 30.6% to 50.7% over a 10‐year period (Fischer et al. 2021). Many reports have shown that coinfection with *S. aureus* and *P. aeruginosa* is more virulent than infection with a single species and worsens the prognosis of patient, especially when both species are multidrug‐resistant strains (Briaud et al. 2019, 2020; Filkins et al. 2015).

Several studies have indicated that bacteria in biofilms are more resistant to antibiotics and host immune defenses than planktonic bacteria (Thöming and Häussler 2022; Moser et al. 2017). *P. aeruginosa* biofilms are frequently found in permanent bladder catheter tubes, ventilator‐associated pneumonia tubes, lungs of patients with CF, and in chronic wounds (Costerton et al. 1999; Cangui‐Panchi et al. 2022; Werneburg 2022). Elimination of these biofilms is difficult because the extracellular polymers within the biofilms inhibit the penetration of antimicrobial agents. Therefore, repeated exposure to high concentrations of antibiotics is required to eliminate biofilms, which increases the risk of the emergence of antibiotic‐resistant strains (Srinivasan et al. 2021; Pinheiro et al. 2014; Dan et al. 2023; Liu et al. 2022). Biofilms are composed of microbial cells and extracellular polymeric substances, including polysaccharides (Di Martino 2018; Donlan 2002). To form biofilm, *P. aeruginosa* synthesizes three types of exopolysaccharides: pellicle (Pel), polysaccharide synthesis locus (Psl), and alginate. Overproduction of alginate promotes *P. aeruginosa* to form persistent infections in the CF lungs, while the rugose colonies overproducing Psl and Pel exopolysaccharides show an enhancement of *P. aeruginosa* persistence in both CF lungs and chronic wounds (Hentzer et al. 2001; Pestrak et al. 2018). There is evidence that Psl exopolysaccharides protect *P. aeruginosa* from host defenses during the initial phase of infection in the CF lungs. Therefore, the Psl‐derived exopolysaccharides in biofilm matrix may be important in the early stage of chronic lung infection before the bacteria change to produce an alginate‐based biofilm matrix (Mishra et al. 2012; Jones and Wozniak 2017).

As mentioned, Psl is one of the exopolysaccharides that plays an important role in *P. aeruginosa* biofilm formation. These saccharides consist of repeating pentasaccharide units of d‐mannose, d‐glucose, and l‐rhamnose. The *psl* gene cluster contains 15 genes (*pslA*–*pslO*), which encode proteins involved in polysaccharide biosynthesis (Byrd et al. 2009). The promoter of *psl* operon is located upstream of the *pslA* gene. Five *psl* genes (*pslA*, *pslE*, *pslJ*, *pslK,* and *pslL*) encoded Psl proteins (PslA, PslE, PslJ, PslK, and PslL, respectively) have inner membrane‐spanning domains and make up the Psl polymerization complex (Franklin et al. 2011). PslA plays a role in initiating Psl polysaccharide synthesis, which is crucial for the early stage of biofilm formation, attachment, and immune evasion. Comparative attachment analysis showed that *P. aeruginosa* PAO1Δ*pslA* is defective in attachment and that constitutive expression of the *psl* operon is required for efficient attachment to surfaces via synthesized exopolysaccharides (Overhage et al. 2005). PslE is involved in the export and assembly of the Psl polysaccharide, contributing to the biofilm's structural integrity, stability, and ability to withstand hostile environments (Franklin et al. 2011). The other three Psl proteins (PslJ, PslK, and PslL) are related to the transport, export, modification, and assembly of the Psl polysaccharide in *P. aeruginosa*. These genes work together to contribute to the biofilm formation and pathogenicity of *P. aeruginosa* to persist in chronic infections (Franklin et al. 2011).

Our previous study has demonstrated that *S. aureus*‐derived extracellular vesicles (SaEVs) promoted *P. aeruginosa* pathogenicity by increasing lipopolysaccharide (LPS) production, biofilm formation, and epithelial cell invasion, and by decreasing the uptake of *P. aeruginosa* by macrophages (Subsomwong et al. 2024). In addition, differential proteomic analysis revealed a significant increase in PslE production in *P. aeruginosa* after SaEV treatment (14‐fold increase compared to non‐SaEV treatment) (Subsomwong et al. 2024). However, the role of PslE and Psl exopolysaccharide polymerization on SaEV‐mediated *P. aeruginosa* pathogenicity is unclear. In this study, we aimed to investigate whether SaEVs influence *P. aeruginosa* pathogenicity in a PslE‐dependent manner. We constructed a *pslE* gene‐deletion mutant of *P. aeruginosa* (PaΔ*pslE*). Using this mutant, the effects of SaEVs on the expression of five genes related to Psl polymerization, biofilm formation, and various aspects of *P. aeruginosa* pathogenicity were investigated in comparison to the *P. aeruginosa* wildtype (PaWT).

## 2 Materials and Methods

### 2.1 Bacterial Strains, Cell Cultures, and Growth Conditions

*P. aeruginosa* strain ATCC 15692, a standard reference strain of the PAO1 lineage, was used in this study. It was cultured in Tryptic soy agar (TSA) (BD Bioscience, Sparks, MD) at 37°C for 24 h. A single colony of *P. aeruginosa* was precultured in Tryptic soy broth (TSB) (BD Bioscience) under aerobic conditions (125 rpm) for 16 h. The *P. aeruginosa* preculture was inoculated into TSB and cultured at 37°C under aerobic conditions for 4 h. Then, the bacterial cells were collected and washed with 1× phosphate‐buffered saline (PBS) twice. The bacterial number was adjusted by converting optical density at 600 nm (OD_600nm_) measured with a UV‐1900i spectrophotometer (Shimadzu, Kyoto, Japan). The bacterial number at OD_600nm_ of 1.0 was estimated to 1.5 × 10^9^ CFU/mL.

HaCaT human keratinocytes and RAW 264.7 mouse macrophages were cultured at 37°C, 5% CO_2_ in Dulbecco's Eagle's minimum essential medium (DMEM; Nissui Pharmaceutical Co., Tokyo, Japan) supplemented with 10% fetal bovine serum (FBS; JRH Biosciences, Lenexa, KS), 0.075% NaHCO_3_ (Wako Pure Chemical Industries Ltd., Osaka, Japan), 0.03% l‐glutamine (Wako Pure Chemical), and 1× Antibiotic–Antimycotic (Gibco; Thermo Fisher Scientific, Waltham, MA).

### 2.2 Construction of *pslE* Gene Deletion in *P. aeruginosa*

**Figure A1:**
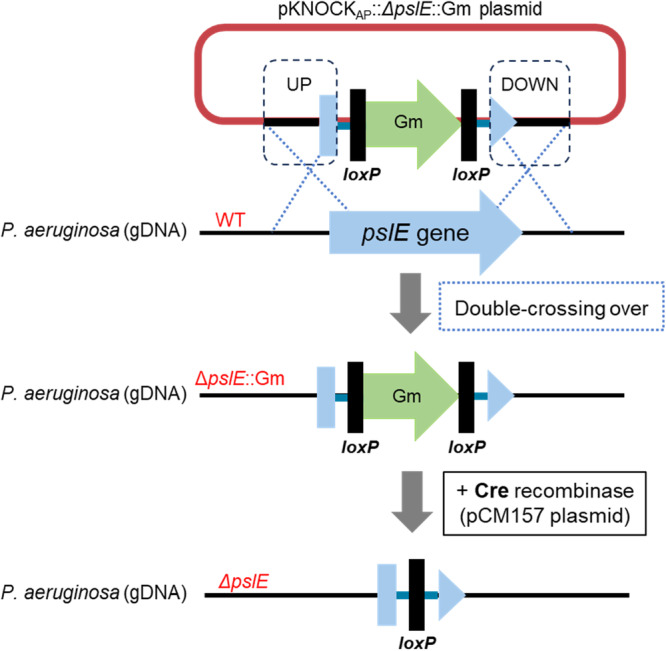
Scheme of double‐crossing over and *loxP*‐Gm‐*loxP* replacement of *pslE* gene in *Pseudomonas aeruginosa* genomic DNA (gDNA) for constructing a *pslE* gene‐deletion mutant of *P. aeruginosa* (Δ*pslE*) from *P. aeruginosa* wildtype (WT) by Cre‐*loxP* system. Gm, gentamicin.

**Figure A2:**
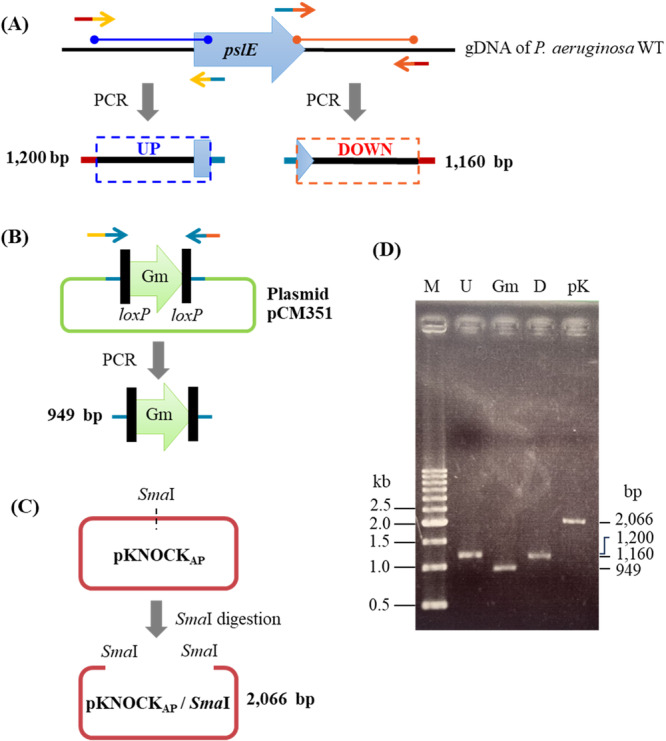
Preparation of DNA fragments for constructing pKNOCK_AP_::Δ*pslE*::Gm plasmid. (A) The upstream region (1200 bp, blue) and downstream region (1160 bp, red) of *pslE* gene in *Pseudomonas aeruginosa* wildtype (WT) were amplified using primer Up_F/Up_R and primer Down_F/Down_R, respectively. (B) The *loxP*‐Gm‐*loxP* fragment (949 bp) was amplified from plasmid pCM351 using primers lxP‐G‐lxP_F and lxP‐G‐lxP_R. (C) The pKNOCK_AP_ plasmid was linearized with *Sma*I (2066 bp). (D) The DNA fragments were purified and checked by 1% gel electrophoresis. M, 500 bp DNA ladder; U, upstream fragment; Gm, *lox*P‐Gm‐*lox*P; D, downstream fragment; pK, linearized pKNOCK_AP_ plasmid. gDNA, genomic DNA; Gm, gentamicin; PCR, polymerase chain reaction.

**Figure A3:**
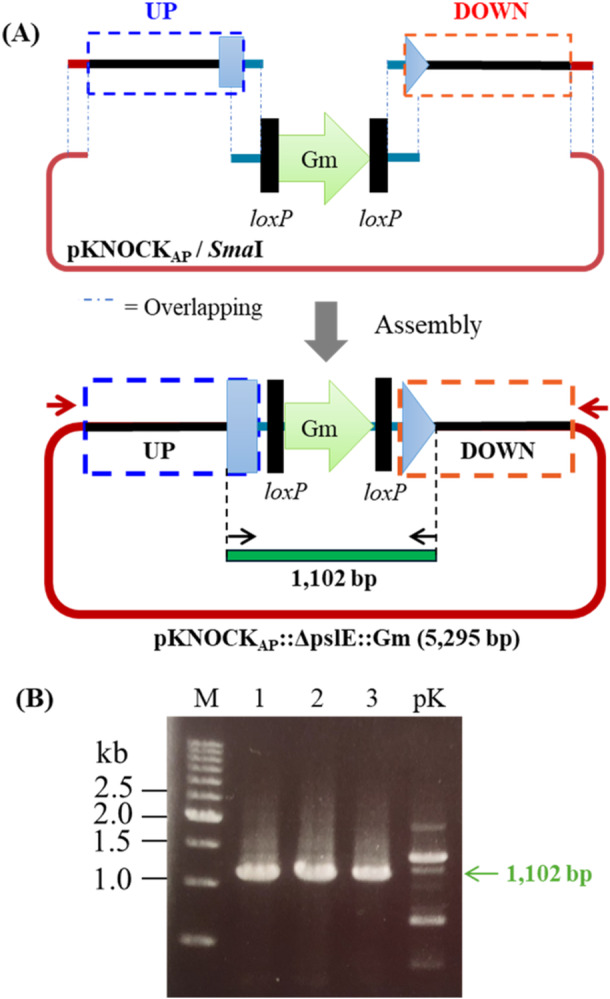
Construction of pKNOCK_AP_::Δ*pslE*::Gm plasmid. (A) Four DNA fragments from Figure A2 were assembled and transformed into *Escherichia coli* BW20767. Transformants were selected on Luria–Bertani agar containing 100 μg/mL ampicillin and 10 µg/mL gentamicin. (B) Three clones (1–3) were obtained as confirmed by PCR using primers *pslE*_F and *pslE*_R with the expected size of PCR product at 1102 bp. The PCR product from pKNOCK_AP_ (pK) was used as a control. M, 500 bp DNA ladder. Gm, gentamicin; PCR, polymerase chain reaction.

**Figure A4:**
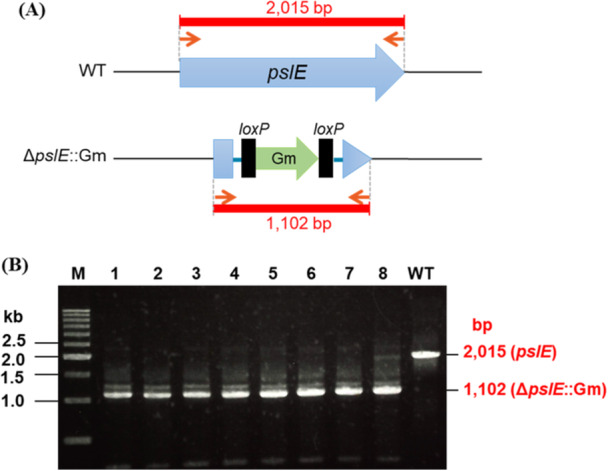
Generation of *Pseudomonas aeruginosa* Δ*pslE*::Gm. Plasmid pKNOCK_AP_::Δ*pslE*::Gm was transformed into *P. aeruginosa* wildtype (WT), and the transformants were selected on Luria–Bertani agar containing 30 μg/mL gentamicin. (A) Scheme of *pslE* gene in *P. aeruginosa* WT and replacement of *pslE* gene with *loxP*‐Gm‐*loxP* in *P. aeruginosa* Δ*pslE*::Gm. The expected sizes of PCR products using primer *pslE*_F and *pslE*_R in *P. aeruginosa* WT and Δ*pslE*::Gm was 2015 and 1102 bp, respectively. (B) Eight Gm‐resistant clones were obtained and the PCR product indicated that *pslE* gene was replaced with *loxP*‐Gm‐*loxP* fragment in all clones. M, 500 bp DNA ladder. Gm, gentamicin; PCR, polymerase chain reaction.

**Figure A5:**
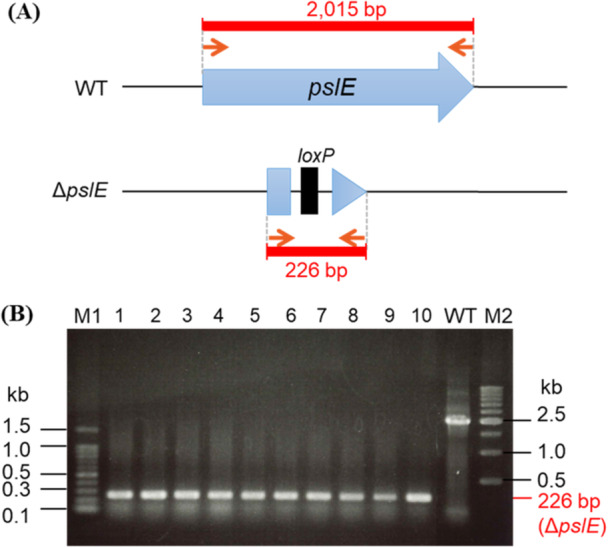
Generation of *Pseudomonas aeruginosa* Δ*pslE*. Plasmid pCM157 was transformed into *P. aeruginosa* Δ*pslE*::Gm, and the transformants were selected on Luria–Bertani agar containing 200 μg/mL tetracycline. On the basis of the activity of Cre recombinase from pCM157 plasmid and *loxP* sequence, Gm‐resistant gene is removed. (A) Scheme of *pslE* gene in *P. aeruginosa* wildtype (WT) and remaining *pslE* gene in *P. aeruginosa* Δ*pslE*. The expected sizes of PCR products using primer *pslE*_F and *pslE*_R in *P. aeruginosa* WT and Δ*pslE* was 2015 and 226 bp, respectively. (B) Ten tetracycline‐resistant clones were randomly selected and the PCR product indicated that *pslE* and Gm‐resistant genes were successfully deleted in all clones. M1 and M2, 100 and 500 bp DNA ladders, respectively. Gm, gentamicin; PCR, polymerase chain reaction.

**Figure A6:**
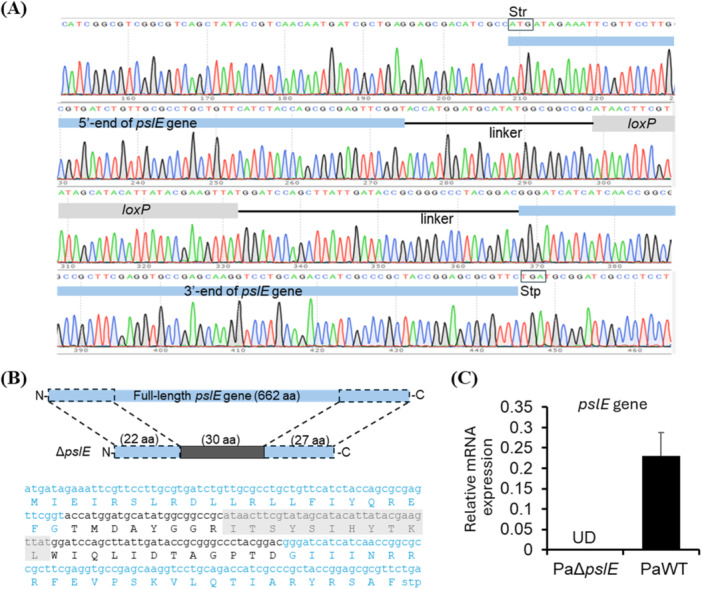
DNA sequence and predicted amino acids after deletion of *pslE* gene in PaΔ*pslE*. (A) DNA sequence demonstrates that 5’‐ and 3’‐ends of *pslE* gene are joined by linkers and *loxP*. Boxes indicate start and stop codon of *pslE* gene. (B) Scheme and predicted amino acid sequence analyzed by Expasy translate tool. Truncated PslE protein consists of 22 and 27 residues from N‐ and C‐terminal of PslE, respectively (blue letters) and 30 residues from linkers and *loxP* (black letters, *loxP* is highlighted in grey). (C) Relative mRNA expression of *pslE* gene in *Pseudomonas aeruginosa* PaΔ*pslE* and wildtype (PaWT) determined by RT‐qPCR neutralizing with expression of *rpoD* gene. The values are from two‐independent experiment (*n* = 6). mRNA, messenger RNA; PCR, polymerase chain reaction; RT‐qPCR, reverse transcription quantitative polymerase chain reaction; UD, undetectable.

Cre‐*loxP* system was used to construct a PaΔ*pslE*. Details of the construction process are provided in Appendix 1 (Tables A1, A2 and Figures A1, A2, A3). The absence of *pslE* gene in the PaΔ*pslE* genome was confirmed by PCR (Figures A4 and A5), DNA sequencing, and reverse transcription (RT)‐qPCR (Figure A6).

### 2.3 Preparation of SaEVs

SaEVs were prepared as described in our previous study (Subsomwong et al. 2024). Briefly, a standard laboratory strain of *S. aureus* (ATCC 1718) was cultured in Brain Heart Infusion broth for 8 h under aerobic conditions. The supernatant was collected and centrifuged to precipitate SaEVs at 100,000*g*, 4°C for 90 min using an ultracentrifuge (Himac CP80WX, HITACHI, Tokyo, Japan). After washing with ice‐cold PBS, the pellet was suspended and subjected to discontinuous iodixanol gradient ultracentrifugation (40%, 20%, 10%, and 5% iodixanol in 0.25 M sucrose/10 mM Tris, pH 7.5). After spinning at 100,000*g*, 4°C for 16 h using a swing out rotor, six fractions were collected from the top of the gradient. The substances in each fraction were then precipitated by ultracentrifugation at 100,000*g* for 2 h at 4°C. The resulting pellets were washed and resuspended in an appropriate volume of ice‐cold PBS. The protein concentration of each fraction was determined by Bradford protein assay (Bio‐Rad Laboratories, Richmond, CA).

### 2.4 Expression Analysis of *Psl*‐Related Genes in *P. aeruginosa* by RT‐qPCR

PaWT and PaΔ*pslE* were precultured and the bacterial number was adjusted to OD_600nm_ = 1.0 (estimated to 1.5 × 10^9^ CFU/mL) in TSB. The bacterial cells were incubated at 37°C for 4 h with or without 5 µg/mL SaEVs under static conditions. The bacterial cells were harvested by centrifugation at 5800*g*, 4°C for 10 min, and washed twice with ice‐cold PBS.

Total RNA was extracted by TRIzol reagent (Thermo Fisher Scientific) as described in the manufacturer's instructions. To degrade the remaining gDNA, DNase I (5 µL of 1 U/µL) (Takara Bio Inc., Shiga, Japan) was added, and the RNA was extracted by TRIzol reagent again. The concentration of RNA was measured using NanoDrop Lite Plus spectrophotometer (Thermo Fisher Scientific). cDNA was synthesized from total RNA, random primer, and M‐MLV reverse transcriptase (Invitrogen). The expression of five genes; *pslA*, *pslE*, *pslJ*, *pslK*, and *pslL*, which are associated with polysaccharide polymerization in *P. aeruginosa psl* operon was determined by RT‐qPCR using SYBR Green Supermix (Bio‐Rad). The expression of *rpoD* gene, encoding RNA polymerase sigma factor, was used for normalization. The primer sequences and RT‐qPCR conditions used in this experiment are mentioned in our previous study (Subsomwong et al. 2024). The cycle threshold (C_T_) values obtained by the Bio‐Rad CFX96 RT‐PCR system were used to calculate the gene expression level. The C_T_ value of the *rpoD* gene was used for normalization with the formula [2^−(CT of target gene − CT of *rpoD* gene)^].

### 2.5 LPS Isolation and Detection

PaWT and PaΔ*pslE* (OD_600nm_ = 1.0) were incubated in TSB with or without 5 µg/mL SaEVs at 37°C for 24 h under static conditions. The bacterial cells were then harvested by centrifugation at 5800*g*, 4°C for 10 min, washed with ice‐cold PBS twice, and stored at −80°C until use.

LPS was extracted from the bacterial cells by a hot aqueous‐phenol method as described previously (Jackson et al. 2004) with some modifications. Briefly, the bacterial cells were resuspended in 200 µL of SDS‐lysis buffer (4% β‐mercaptoethanol, 4% SDS, and 20% glycerol in 0.1 M Tris‐HCl, pH 6.8) and boiled for 15 min. DNase I and RNase solution (5 µL of 10 mg/mL each; Roche Diagnostic, Mannheim, Germany) were added and the sample was incubated for 30 min at 37°C to eliminate the DNA and RNA, respectively. Thereafter, proteinase K (10 µL of 10 mg/mL; Roche Diagnostic) was added and the sample was incubated at 59°C overnight. Ice‐cold Tris‐saturated phenol (200 µL; Invitrogen) was then added, the sample was incubated at 65°C for 15 min, cooled down to room temperature, and vortexed for 5–10 s with diethyl ether (1 mL; Nacalai Tesque Inc., Kyoto, Japan). Blue solution in the bottom layer was collected after centrifugation at 20,600*g* for 10 min. LPS in the sample was re‐extracted with the ice‐cold Tris‐saturated phenol step until the solution in the bottom layer was clear. The purified LPS was observed by SDS‐PAGE and stained using a Silver Stain 2 kit (Wako Pure Chemical) according to manufacturer's instructions. The quantitative intensity of each group was measured using ImageJ 1.54 g software, and the intensity of PaWT without SaEVs was set as 1.00.

### 2.6 Biofilm Formation and Quantification

PaWT and PaΔ*pslE* were adjusted to OD_600 nm_ = 1.0 in TSB with or without 5 μg/mL SaEVs and were transferred to 96‐well plates (100 μL/well). After incubation at 37°C for 16 h, the planktonic cells in the culture supernatant were then removed. The biofilm was fixed by adding absolute methanol and incubating at room temperature for 15 min. The methanol was removed, the plates were left to dry at room temperature. The biofilm was stained with crystal violet solution [1% (w/v) in distilled water] for 5 min, washed with distilled water twice, and dried. Crystal violet bound to biofilm was dissolved in 96% ethanol and quantified by measuring the OD at 595 nm using a microplate reader (MULTISCAN Sky, Thermo Fisher Scientific). The relative biofilm biomass was calculated based on the biomass of PaWT without SaEVs as 100%.

### 2.7 Invasion of *P. aeruginosa* Into Human Keratinocytes

HaCaT cells (5 × 10^6^ cells/well) were seeded in 24‐well plates and incubated at 37°C, 5% CO_2_ for 48 h. Meanwhile, PaWT and PaΔ*pslE* were prepared and adjusted to OD_600 nm_ = 1.0 in TSB. After incubation with or without 5 µg/mL SaEVs at 37°C for 24 h, the bacterial cells were collected, washed with PBS twice, and resuspended in antibiotic‐free DMEM. The HaCaT cells were washed with washing medium (FBS‐ and antibiotic‐free DMEM) and infected with PaWT or PaΔ*pslE* at MOI = 100. After incubation at 37°C, 5% CO_2_ for 90 min, extracellular bacterial cells in the culture supernatant were removed. The cells were then washed twice with washing medium, once with PBS, and incubated in washing medium containing 120 μg/mL of Gm (Wako Pure Chemical) for 1 h. The cells were washed twice with washing medium and once with PBS, and then lysed with 1% 3‐[(3‐cholamidopropyl)‐dimethylammonio]−1‐propanesulfonate (DOJINDO, Kumamoto, Japan) in PBS for 15 min. Intracellular bacterial number was evaluated by plate count assay after incubating the TSA plates at 37°C for 24 h.

### 2.8 *P. aeruginosa* Uptake by Macrophages

RAW 264.7 cells (1 × 10^6^ cells/well) were seeded into 24‐well plates and incubated at 37°C, 5% CO_2_ for 48 h. Meanwhile, PaWT and PaΔ*pslE* were adjusted, incubated with or without 5 µg/mL SaEVs for 24 h, and collected as described above. The RAW 264.7 cells were washed with washing medium and infected with PaWT or PaΔ*pslE* at MOI = 10. After incubation at 37°C, 5% CO_2_ for 90 min, extracellular bacterial cells in culture supernatant were eliminated, and intracellular bacterial cells were evaluated by plate count assay.

### 2.9 Statistical Analysis

Statistical analyses were performed using GraphPad Prism (version 10.0; GraphPad Software Inc., La Jolla, CA). Data were presented as mean ± standard deviation, mode, min, and max. One‐way ANOVA with Tukey's multiple comparison test was used for relative gene expression analysis. Kruskal–Wallis with Dunn's multiple comparison test was applied for biofilm formation, bacterial uptake by macrophages, and bacterial invasion to keratinocytes experiments. The data are considered statistically significant when a *p* value is < 0.05. Significant levels indicating statistical significance were denoted as follows: *p < 0.05, **p < 0.01, ***p < 0.001, and ****p < 0.0001.

## 3 Results

### 3.1 SaEVs Enhance the Expression of Polysaccharide Polymerization‐Related Genes in the *P. aeruginosa psl* Cluster via *pslE* Gene

**Figure 1:**
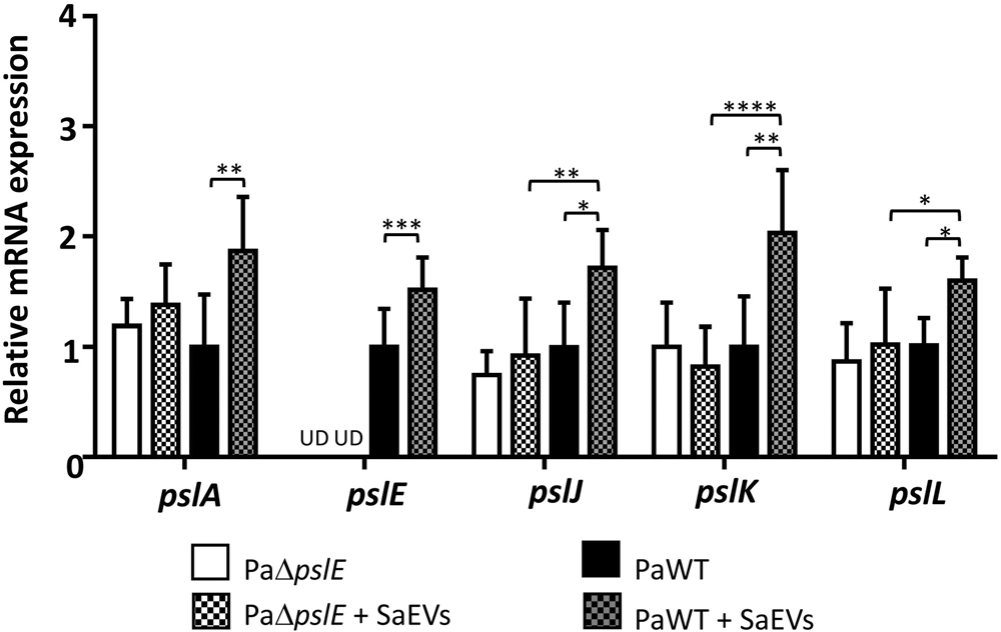
Effect of SaEVs on the expression of polysaccharide polymerization‐related genes in *psl* cluster of *pslE* gene‐deletion mutant of *P. aeruginosa* (PaΔ*pslE*) and *P. aeruginosa* wildtype (PaWT). PaΔ*pslE* and PaWT were treated with 0 and 5 µg/mL SaEVs for 4 h. Relative mRNA expression of *pslA, pslE, pslJ, pslK*, and *pslL* was assessed by RT‐qPCR. The values are from two independent experiments (*n* = 6). Relative gene expression was calculated by referring to the expression of each target gene in PaWT as 1.0. Statistical analysis was performed using one‐way ANOVA with Tukey's multiple comparison test for relative gene expression. Asterisks indicate *p* values (*p < 0.05, **p < 0.01, ***p < 0.001, ****p < 0.0001). UD: undetectable.

PaΔ*pslE* was constructed, and successful deletion of the *pslE* gene was confirmed (Figure A6). By using this mutant and PaWT, we investigated whether SaEVs promote *P. aeruginosa* pathogenicity via PslE and Psl polysaccharide polymerization. The effect of SaEVs on the expression of *pslE* and other Psl polysaccharide polymerization‐related genes in *psl* cluster was evaluated. As shown in Figure 1, SaEVs significantly upregulated the expression of all five polysaccharide polymerization‐related genes in the *psl* cluster of PaWT. Based on the *pslE* gene deletion in the PaΔ*pslE* genome, *pslE* gene expression in this PaΔ*pslE* mutant was undetectable. In addition, the ability of SaEVs to promote the expression of *pslA*, *pslJ*, *pslK*, and *pslL* genes in the PaΔ*pslE* was abolished (Figure 1). These results suggest that SaEVs enhance the expression of polysaccharide polymerization‐related genes, and the presence of *pslE* gene is required for SaEVs to promote *pslA*, *pslJ*, *pslK*, and *pslL* gene expression.

### 3.2 SaEVs Enhance LPS Production in *P. aeruginosa* Partially in a *pslE*‐Dependent Manner

**Figure 2:**
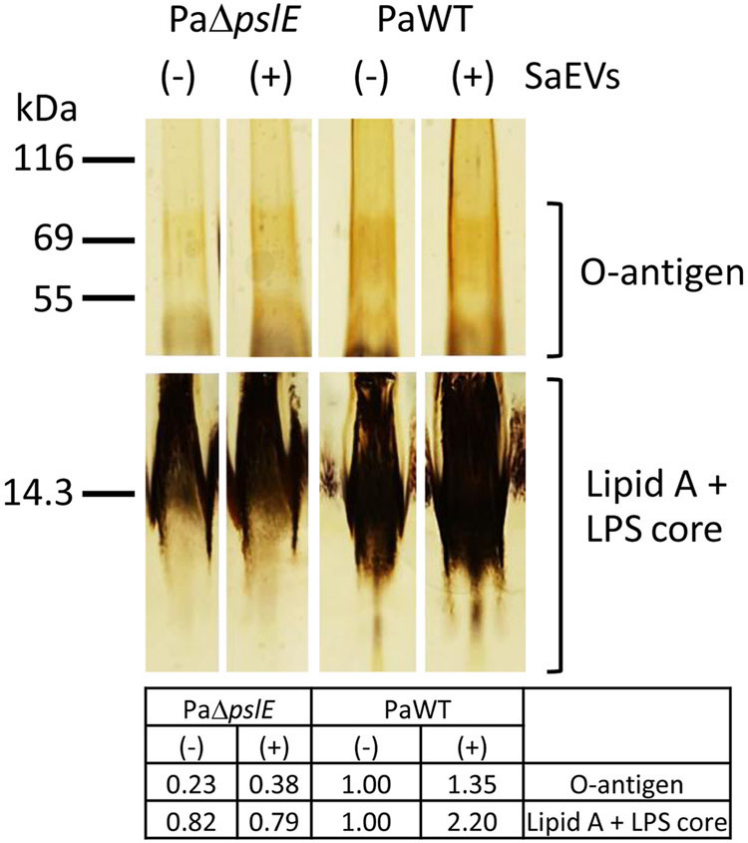
Effect of SaEVs on LPS production in *pslE* gene deletion mutant of *P. aeruginosa* (PaΔ*pslE*) and *P. aeruginosa* wildtype (PaWT). PaΔ*pslE* and PaWT were treated with or without 5 µg/mL SaEVs for 24 h. LPS were isolated, separated by SDS‐PAGE, and stained using Silver Stain kit. All samples were started with the same number of bacterial cells, prepared by the same process, and applied in equal volumes. The table demonstrates the quantitative intensity of each group, setting the intensity of PaWT without SaEVs as 1.00.

Since the function of *psl* gene cluster is involved in biofilm formation (Jones and Wozniak 2017), and LPS has been implicated in the architecture of *P. aeruginosa* biofilm (Rocchetta et al. 1999), we speculated that SaEVs may affect LPS production via *pslE* upregulation. Therefore, we further examined the ability of SaEVs to promote LPS production in PaΔ*pslE* and PaWT. As shown in Figure 2, SaEVs‐treated PaWT had a higher intensity of O‐antigen (1.35‐fold) and lipid A and LPS core (2.20‐fold) compared to untreated PaWT control. LPS profile in PaΔ*pslE* was identical to that in PaWT, although deletion of *pslE* gene resulted in a reduced intensity of O‐antigen (0.23‐fold) and lipid A and LPS core (0.82‐fold). In the PaΔ*pslE*, the activity of SaEVs to promote lipid A and LPS core was abolished (0.79‐fold). However, SaEVs still promoted O‐antigen intensity (0.38‐fold) in the PaΔ*pslE* (Figure 2). These results suggest that SaEVs may promote the LPS production in *P. aeruginosa* partially via *pslE* gene. In particular, SaEVs are involved in lipid A and LPS core but not O‐antigen biosynthesis in the *pslE*‐dependent manner.

### 3.3 *pslE* Gene is Required for SaEVs to Promote Biofilm Formation in *P. aeruginosa*

**Figure 3:**
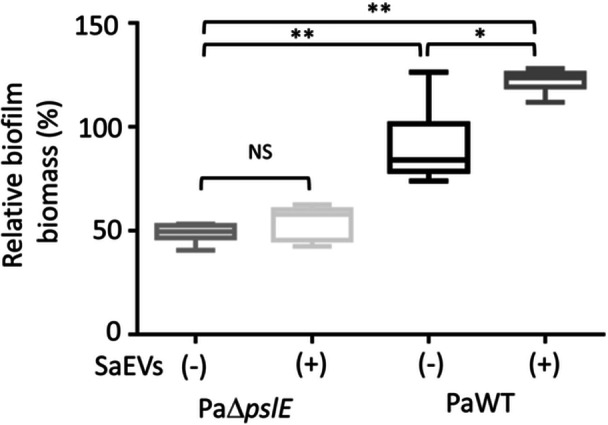
Effect of SaEVs on biofilm formation in *pslE* gene deletion mutant of *P. aeruginosa* (PaΔ*pslE*) and *P. aeruginosa* wildtype (PaWT). PaΔ*pslE* and PaWT were cultured with or without 5 µg/mL SaEVs for 16 h. Biofilm biomass was quantified by crystal violet assay. Relative biofilm biomass was calculated by referring PaWT without SaEVs as 100%. The values are from two independent experiments (*n* = 6). Statistical analysis was performed using Kruskal–Wallis with Dunn's multiple comparisons. Asterisks indicate *p* values (*p < 0.05, **p < 0.01), NS: not significant.

We further investigated the effect of SaEVs on the biofilm formation in PaΔ*pslE* and PaWT. As expected, SaEVs significantly enhanced the biofilm biomass of PaWT (122.0%) compared to untreated PaWT (Figure 3). In addition, the biofilm biomass of PaΔ*pslE* was significantly reduced by ~50% compared to that of PaWT. There was no significant difference in biofilm biomass between SaEV‐treated and untreated PaΔ*pslE* (Figure 3). These results suggest that *pslE* is one of the important factors for biofilm formation in *P. aeruginosa* and SaEVs promote biofilm formation through the *pslE* gene. In addition, the biofilm biomass in PaΔ*pslE* may be formed by other mechanisms that are not influenced by SaEVs.

### 3.4 SaEVs and *pslE* Gene Are Not Involved in *P. aeruginosa* Invasion Into Epithelial Cells

**Figure 4:**
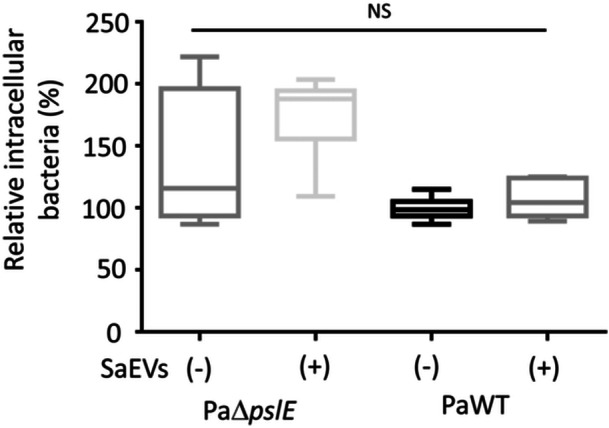
Effect of SaEVs on invasion of *pslE* gene deletion mutant of *P. aeruginosa* (PaΔ*pslE*) and *P. aeruginosa* wildtype (PaWT) into human keratinocytes. PaWT and PaΔ*pslE* were treated with or without 5 µg/mL SaEVs for 24 h before infection. HaCaT cells were infected with PaΔ*pslE* and PaWT at MOI = 100 for 90 min. The extracellular bacterial cells were eliminated by gentamicin treatment for 1 h. Then, the intracellular bacterial number was evaluated by plate count assay. Relative intracellular bacterial number was calculated by referring PaWT without SaEVs as 100%. The values are from two independent experiments (*n* = 6). Statistical analysis was performed using Kruskal–Wallis with Dunn's multiple comparisons. NS: not significant.

We have previously demonstrated that SaEVs promote *P. aeruginosa* invasion into human keratinocyte HaCaT cells (Subsomwong et al. 2024). However, we were unable to replicate this result in the current experiment. As shown in Figure 4, intracellular bacterial numbers of SaEV‐treated and untreated PaWT were comparable. Although the intracellular bacterial number of PaΔ*pslE* was higher than that of PaWT, this difference was not statistically significant in our data. In addition, pretreatment of PaΔ*pslE* with SaEVs did not affect the intracellular bacterial number of PaΔ*pslE* in the HaCaT cells (Figure 4). This result suggests that SaEVs and *pslE* gene do not contribute to *P. aeruginosa* invasion into the epithelial cells.

### 3.5 SaEVs Reduce Uptake of *P. aeruginosa* by Macrophages in a *pslE*‐Dependent Manner

**Figure 5:**
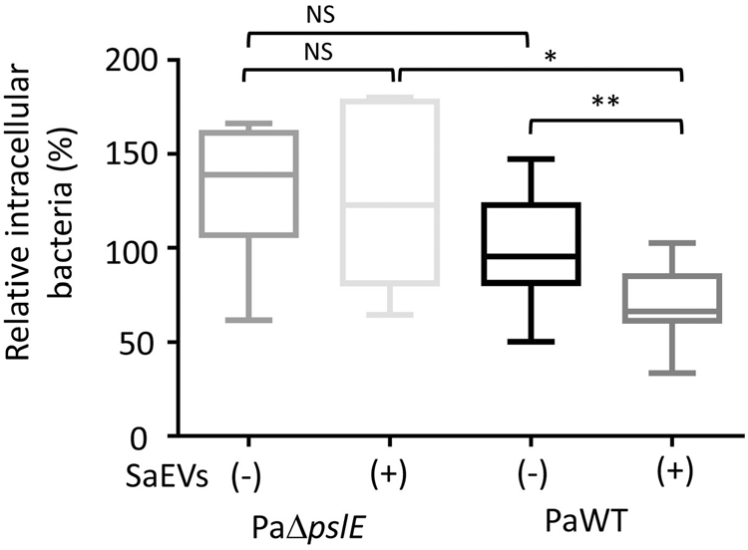
Effect of SaEVs on uptake of *pslE* gene deletion mutant of *P. aeruginosa* (PaΔ*pslE*) and *P. aeruginosa* wildtype (PaWT) by mouse macrophages. PaΔ*pslE* and PaWT were treated with or without 5 µg/mL SaEVs for 24 h before infection. RAW 264.7 cells were infected with PaΔ*pslE* and PaWT at MOI = 10 for 90 min. The extracellular bacterial cells were eliminated by gentamicin treatment for 1 h. Then, the intracellular bacterial number was evaluated by plate count assay. Relative intracellular bacterial number was calculated by referring PaWT without SaEVs as 100%. The values are from two independent experiments (*n* = 6). Statistical analysis was performed using Kruskal–Wallis with Dunn's multiple comparisons. Asterisks indicate *p* values (*p < 0.05, **p < 0.01), NS: not significant.

Biofilm formation is a key factor in *P. aeruginosa* resistance to host immune defenses (Thöming and Häussler 2022; Moser et al. 2017). Therefore, we further investigated whether *pslE* upregulation in *P. aeruginosa* by SaEVs contributes to evasion of macrophage uptake. Our results demonstrated that the uptake of PaWT by macrophages significantly reduced by SaEV‐treatment (Figure 5). Lack of *pslE* gene in PaΔ*pslE* resulted in a slight increase of PaΔ*pslE* uptake by macrophages but this increase did not reach statistical significance. In addition, SaEVs did not alter intracellular bacterial number of PaΔ*pslE* in macrophages. These results suggest that SaEVs reduce uptake of *P. aeruginosa* by macrophages in a *pslE*‐dependent manner.

## 4 Discussion and Conclusions

Coinfections with *P. aeruginosa* and *S. aureus* are commonly found in patients with chronic conditions, including CF and chronic wounds (Bernardy et al. 2022; DeLeon et al. 2014; Fugère et al. 2014). *P. aeruginosa* and *S. aureus* can form mixed biofilms that protect them from the host immune defenses and significantly reduce the efficiency of antibiotic treatment, resulting in recalcitrant, difficult‐to‐treat chronic infections (Vestweber et al. 2024), leading to increased morbidity and mortality worldwide (Bernardy et al. 2022; Phan et al. 2023; Hubert et al. 2013; Limoli et al. 2016). The interaction between *P. aeruginosa* and *S. aureus* has been reported. However, their interaction mediated by EVs and the underlying mechanisms are not well understood. Our previous study has reported that EVs secreted by *S. aureus* interact *P. aeruginosa*, promote biofilm formation and contribute to *P. aeruginosa* pathogenicity (Subsomwong et al. 2024). To understand more detailed mechanisms, differential proteomic analysis between SaEV‐treated and untreated *P. aeruginosa* was performed. Our data revealed that 22 proteins were upregulated (Subsomwong et al. 2024). Among these proteins, a 74.56 kDa protein which is identical to PslE in *P. aeruginosa* PAO1 shows the highest fold change with 14.85‐fold (Subsomwong et al. 2024). Since PslE is one of the polysaccharide polymerization proteins in *psl* cluster (Franklin et al. 2011), we speculated that SaEVs may promote Psl exopolysaccharide production via PslE production, resulting in an enhancement of *P. aeruginosa* pathogenicity.

The results in this study demonstrated that SaEVs significantly upregulated the expression of *pslE* gene as well as the other polysaccharide polymerization‐related genes (*pslA*, *pslJ*, *pslK*, and *pslL*) in *psl* operon of PaWT. Interestingly, the effect of SaEVs in promoting *pslA*, *pslJ*, *pslK*, and *pslL* gene expression was abolished in PaΔ*pslE*. This result suggests that SaEVs may not directly regulate the expression *pslA*, *pslJ*, *pslK*, and *pslL* gene, but rather these genes may be upregulated by SaEVs through *pslE* gene or PslE protein. Although this mechanism is required to be elucidated, the expression of all five polysaccharide polymerization‐related genes in *psl* operon promoted by SaEVs may act together to enhance exopolysaccharide polymerization in the PaWT.

The phosphomannomutase enzyme (encoded by the *algC* gene), which is crucial for alginate exopolysaccharide biosynthesis, was shown to be involved in LPS biosynthesis (Ye et al. 1994). This enzyme helps to convert mannose‐6‐phosphate to mannose‐1‐phosphate, a precursor for both alginate and LPS. Jackson et al. (2004) have demonstrated that an *algC* mutant of *P. aeruginosa* PAO1 clearly lacks core oligosaccharides. Although *psl* cluster is not directly involved in the LPS biosynthesis, we determined if the *psl*‐dependent effect of SaEVs is LPS related. Our results showed that the intensity of LPS in PaΔ*pslE* was lower than that in the PaWT. However, the LPS profile in both O‐antigen and LPS core in PaΔ*pslE* was identical to that in PaWT. The activity of SaEVs to promote lipid A and LPS core was abolished in PaΔ*pslE*, suggesting that SaEVs may be involved in lipid A and LPS core biosynthesis via *pslE* gene. However, SaEVs still promoted O‐antigen production in PaΔ*pslE*. These results suggested that the activity of SaEVs in promoting O‐antigen production may be involved in the other mechanism(s) rather than *psl*‐dependent manner.

Exopolysaccharides are important for the formation and stability of biofilms and the biofilm benefits *P. aeruginosa* by promoting its survival in stressful environments and allowing its persistence on the surface (Ciofu and Tolker‐Nielsen 2019; Thi et al. 2020). The role of Psl in mucoid biofilm has been reported. The ability of *P. aeruginosa* in forming biofilms decreased when the Psl‐encoding genes are absent, indicating that Psl is a crucial component for mucoid biofilm formation (Ma et al. 2012). Disruption of the initial gene of the *psl* cluster (*pslA* either alone or with *pslB*) in *P. aeruginosa* PAO1 affects biofilm formation under both static and continuous flow culture conditions (Overhage et al. 2005; Jackson et al. 2004). Likewise, the mutation of PA2231 (*pslA*) in the PAO1 strain resulted in the formation of thin, unstructured, abnormal biofilms that differ from those formed by the wildtype strain (Jackson et al. 2004; Matsukawa and Greenberg 2004). Our result showed that PaΔ*pslE* exhibited about 50% reduction in biofilm biomass compared to the PaWT. This suggests that not only *pslA* and *pslB*, but also *pslE* plays a role in biofilm synthesis. In addition, SaEVs upregulate *pslE* gene expression, resulting in an enhancement of biofilm formation.

Evasion of phagocytosis by host immune cells is one of the pathogenic mechanisms used by *P. aeruginosa* to persist in the host. Phagocytosis by innate immune cells is a process by which the host cells eradicate the microbes (Hastings et al. 2023; Marzhoseyni et al. 2023). Exopolysaccharides on the surface of *P. aeruginosa* are not only important for biofilm formation but also have been known to play a significant role in reducing immune cell recognition and uptake (Leid et al. 2005). It has been reported that Psl‐deficient *P. aeruginosa* were internalized and killed by neutrophils and macrophages more efficiently than PaWT and Psl overexpressing strains (Mishra et al. 2012). Likewise, our results demonstrated that SaEVs reduce *P. aeruginosa* uptake by macrophages in a *pslE*‐dependent manner. Regarding epithelial cell invasion, our data suggest that neither SaEVs nor *pslE* gene contribute to *P. aeruginosa* invasion into the keratinocytes. We hypothesized that the bacteria produce proteins related to cell invasion, host immune response, and colonization during the acute phase, while proteins associated with biofilm formation, antibiotic resistance, and immune evasion are produce during chronic phase. Fleming et al. (2022) showed that neither the overexpression nor the absence of Pel and Psl polysaccharides significantly alter the ability of *P. aeruginosa* to establish an infection and survive in a mouse model of wound infection. These data suggest that the production of Psl exopolysaccharides does not affect the pathogenesis of *P. aeruginosa* in keratinocytes and wound infections.

Overall, our results demonstrated that SaEVs promote gene expression in *psl* cluster, biofilm formation, and antiphagocytosis of *P. aeruginosa* in a *pslE‐*dependent manner. However, the detailed mechanism by which SaEVs regulate the pathogenicity of *P. aeruginosa* via the *psl* operon remains unknown. We hypothesized that SaEVs may activate the *psl* promoter and/or directly upregulate the *pslE* gene to promote the expression of other *psl* genes and exopolysaccharide production. In addition, the molecules in SaEVs involved in the regulation of *psl* operon need to be considered.

In conclusion, the present study demonstrated that EVs derived from *S. aureus* mediate the pathogenicity of *P. aeruginosa* via a *pslE*‐dependent mechanism, resulting in increased biofilm formation and antiphagocytosis by macrophages. These findings are important to support further development of therapeutic approaches against *S. aureus* and *P. aeruginosa* coinfection, such as inhibiting SaEV interaction with *P. aeruginosa* or targeting *psl* polymerization to inhibit biofilm formation. In addition, the knowledge of EV‐mediated interactions and pathogenicity will lead to the development of therapeutics against other coinfections with opportunistic bacteria, especially those capable of forming biofilms.

## Author Contributions

**Phawinee Subsomwong:** conceptualization (supporting), methodology (lead), data curation (lead), investigation (lead), validation (lead), formal analysis (lead), funding acquisition (lead), writing – original draft (lead), writing – review and editing (supporting). **Rojana Sukchawalit:** methodology (supporting), resources (supporting), validation (supporting), writing – review and editing (supporting). **Naoko Watabe:** software (supporting), writing – review and editing (supporting). **Akio Nakane:** funding acquisition (supporting), writing – review and editing (supporting). **Krisana Asano:** conceptualization (lead), supervision (lead), project administration (lead), funding acquisition (supporting), writing – original draft (supporting), writing – review and editing (lead).

## Ethics Statement

The authors have nothing to report.

## Conflicts of Interest

The authors declare no conflicts of interest.

## Data Availability

The data that support the findings of this study are available on request from the corresponding author.

## Appendix 1

**Materials and Methods**

**Construction of** ***pslE*** **Gene Deletion in** ***Pseudomonas aeruginosa***

*P. aeruginosa* strain American Type Culture Collection (ATCC) 15692, *Escherichia coli* BW20767 and *E. coli* DH5α were cultured in Luria–Bertani (LB) medium (Invitrogen, Carlsbad, CA). Plasmids, pCM351 and pCM157, were purchased from Addgene (Watertown, MA). Plasmid pKNOCK_AP_ was kindly provided by associate professor Rojana Sukchawalit, Laboratory of Biotechnology, Chulabhorn Research Institute, Lak Si, Bangkok, Thailand. The plasmids were transformed into *E. coli* and/or *P. aeruginosa* and cultured in appropriate antibiotics (Table A1).

**Table A1:** Bacterial strains and appropriate antibiotics used for constructing PaΔ*pslE*.

Bacterial strain	Appropriate antibiotics in LB broth or LB agar
*Pseudomonas aeruginosa* ATCC 15692	None
*Escherichia coli* DH5α /pCM351	100 µg/mL ampicillin
*E. coli* BW20767	None
*E. coli* BW20767/pKNOCK_AP_	100 µg/mL ampicillin
*E. coli* BW20767/pKNOCK_AP_::Δ*pslE*::Gm	100 µg/mL ampicillin and 10 µg/mL gentamicin
*P. aeruginosa/*pKNOCK_AP_::Δ*pslE*::Gm	30 µg/mL gentamicin
*E. coli* DH5α /pCM157	10 µg/mL tetracycline
*P. aeruginosa*/pCM157	200 µg/mL tetracycline
*P. aeruginosa* Δ*pslE*	None

Abbreviations: ATCC, American Type Culture Collection; LB, Luria–Bertani.

A PaΔ*pslE* mutant was generated by Cre‐*loxP* system (Figure A1). Upstream and downstream regions of *pslE* gene from genomic DNA (gDNA) of *P. aeruginosa* ATCC 15692, and *loxP*‐gentamicin (Gm)‐*loxP* fragment from pCM351 plasmid were amplified by polymerase chain reaction (PCR) using AmpliTaq Gold DNA Polymerase (Thermo Fisher Scientific, Waltham, MA). Sequence for assembly and in‐frame deletion was designed and added to 5’‐end of each primer. The primers used in this study are listed in Table A2. Plasmid pKNOCK_AP_ was linearized with *Sma*I (Takara Bio Inc., Shiga, Japan). To generate pKNOCKAP::Δ*pslE*::Gm plasmid, the PCR fragments were then assembled and ligated with *Sma*I‐linearized pKNOCK_AP_ plasmid using NEB builder HiFi DNA Assembly kit (New England Biolabs, Ipswich, MA). After transformation into *E. coli* BW20767, clones containing the pKNOCKAP::Δ*pslE*::Gm plasmid were confirmed by PCR using *pslE*_F and *pslE*_R primers (Table A2) and DNA sequencing.

**Table A2:** Primers used for constructing PaΔ*pslE*.

Primer sequence (5’–3’)	Amplified fragment	Product size
UP_F: ctagaactagtggatcccccATTCCCACGATTACCGCT UP_R: ccatggtaccGAACTCGCGCTGGTAGATGA	Upstream region of *pslE*	1200 bp
Down_F: gggccctacgGACGGGATCATCATCAACCG Down_R: atatcgaattcctgcagcccCAACAACTGTTCGGAGAGGG	Downstream region of *pslE*	1160 bp
lxP‐G‐lxP_F: gatcatcgtcGGTACCATGGATGCATATGGC lxP‐G‐lxP_R: gtggtgaggaCGTAGGGCCCGCGGTATC	*loxP*‐Gm‐*loxP*	949 bp
*pslE*_F: GGAGCGACATCGCC **ATG** ATA *pslE*_R: GGGCGATCCGCA **TCA** GAA	*pslE* gene (from start to stop codon)	WT: 2015 bp Δ*pslE*::Gm: 1102 bp Δ*pslE*: 226 bp

*Note:* Lower case letters: Sequence for assembly. Bold letter and underlined: Start and stop codons of *pslE* gene.

The obtained pKNOCKAP::Δ*pslE*::Gm plasmid was isolated from *E. coli* BW20767 using QIAGEN plasmid isolation kit (Qiagen, Valencia, CA) and transformed into *P. aeruginosa* ATCC 15692 by electroporation using a MicroPulser Electroporator (Bio‐Rad Laboratories Inc., Hercules, CA). Gm‐resistant colonies of *P. aeruginosa* Δ*pslE*::Gm were selected for confirming double‐crossing over with *loxP*‐Gm‐*loxP* replacement of the *pslE* gene by PCR. To remove Gm gene, pCM157 plasmid for Cre recombinase expression was transformed into the *P. aeruginosa* Δ*pslE*::Gm. From the Gm‐sensitive and tetracycline‐resistant colonies, the removal of Gm gene by Cre recombinase and in‐frame deletion of *pslE* gene was confirmed by PCR and DNA sequencing. Alignments for both DNA and amino acid sequence were performed using MEGA‐X software (v10.0). The *P. aeruginosa* Δ*pslE* was subcultured on LB agar without antibiotics until clean deletion mutant (without pCM157 plasmid) with tetracycline‐sensitive colonies was obtained. The expression level of *pslE* gene in PaΔ*pslE* was evaluated by reverse transcription quantitative polymerase chain reaction (RT‐qPCR).

**Results**

**Construction of PaΔ** ***pslE***

To construct pKNOCK_AP_::Δ*pslE*::Gm plasmid, upstream and down regions of *pslE* gene (1200 and 1160 bp, respectively) were amplified from the gDNA of *P. aeruginosa* ATCC 15692 by PCR (Figure A2A). Simultaneously, *loxP*‐Gm‐*loxP* DNA fragment (949 bp) was amplified from pCM351 plasmid (Figure A2B), and pKNOCK_AP_ plasmid was linearized with *Sma*I (2066 bp) (Figure A2C). All purified DNA fragments were confirmed by 1% agarose electrophoresis (Figure A2D). These fragments were assembled in a 1:1:1:1 ratio and transformed into *E. coli* BW20767. As confirmed by PCR, 3 Gm‐resistant clones containing pKNOCK_AP_::Δp*slE*::Gm were obtained (Figure A3). Plasmids from these clones were purified and proceeded with DNA sequencing. Clone 1 with correct DNA sequence was selected for generating *P. aeruginosa* Δp*slE*::Gm. Plasmid pKNOCK_AP_::Δ*pslE*::Gm from Clone 1 was isolated and transformed into *P. aeruginosa* ATCC by electroporation. Eight Gm‐resistant clones were obtained and the PCR results confirmed that *pslE* gene was replaced with *loxP*‐Gm‐*loxP* fragment in all clones (Figure A4). Clone 3 was selected for removal of Gm‐resistant gene by Cre recombinase. The removal of Gm resistance gene was detected by PCR (Figure A5), and in‐frame deletion of *pslE* gene was confirmed by DNA sequencing (Figure A6A). The result showed that 5’ and 3’ nucleotide sequences of the *pslE* gene were intact, whereas the middle part of the gene was replaced with short linkers and *loxP* sequence. By using DNA translation tool, this sequence translates to a 79‐amino‐acid protein consisting of 22 and 27 amino acids for N‐ and C‐terminal regions of PslE, respectively (Figure A6B). A Protein BLAST analysis provided by the National Center for Biotechnology Information showed no similarity between this protein and other essential proteins of *P. aeruginosa*. Likewise, RT‐qPCR confirmed the successful removal of the *pslE* gene from the PaΔ*pslE* genome, resulting in undetectable *pslE* gene expression in this deletion mutant (Figure A6C).
